# Transcription factor NRF2 as potential therapeutic target for preventing muscle wasting in aging chronic kidney disease patients

**DOI:** 10.1007/s40620-022-01484-w

**Published:** 2022-11-02

**Authors:** Erika F. Gómez-García, Fabiola Martín del Campo, Laura Cortés-Sanabria, Francisco Mendoza-Carrera, Carla Maria Avesani, Peter Stenvinkel, Bengt Lindholm, Alfonso M. Cueto-Manzano

**Affiliations:** 1 Unidad de Investigación Biomédica 02, Hospital de Especialidades, Centro Médico Nacional de Occidente, Instituto Mexicano del Seguro Social, Guadalajara, Mexico; 2 Unidad de Investigación Biomédica 01, Centro de Investigación Biomédica de Occidente, Instituto Mexicano del Seguro Social, Guadalajara, Mexico; 3 Facultad de Medicina y Psicología, Universidad Autónoma de Baja California, Tijuana, Mexico; 4 Renal Medicine and Baxter Novum, Clintec, Karolinska Institutet, M99 Karolinska University Hospital Huddinge, 14186 Stockholm, Sweden

**Keywords:** NRF2, Muscle wasting, Oxidative stress, Inflammation, Chronic kidney disease

## Abstract

Increased muscle protein catabolism leading to muscle wasting is a prominent feature of the syndrome of protein-energy wasting (PEW) in patients with chronic kidney disease (CKD). PEW and muscle wasting are induced by factors such as inflammation, oxidative stress and metabolic acidosis that activate the *ubiquitin–proteasome system*, the main regulatory mechanism of skeletal muscle degradation. Whether deficiency of nuclear factor erythroid 2-related factor 2 (NRF2), which regulates expression of antioxidant proteins protecting against oxidative damage triggered by inflammation, may exacerbate PEW has yet to be examined in aging patients with CKD. This review focuses on the hypothesis that NRF2 is involved in the maintenance of muscle mass and explores whether sustained activation of NRF2 by non-pharmacological interventions using nutraceutical activators to improve redox homeostasis could be a plausible strategy to prevent skeletal muscle disorders, including muscle wasting, sarcopenia and frailty associated with PEW in aging CKD patients.

**Graphical abstract:**

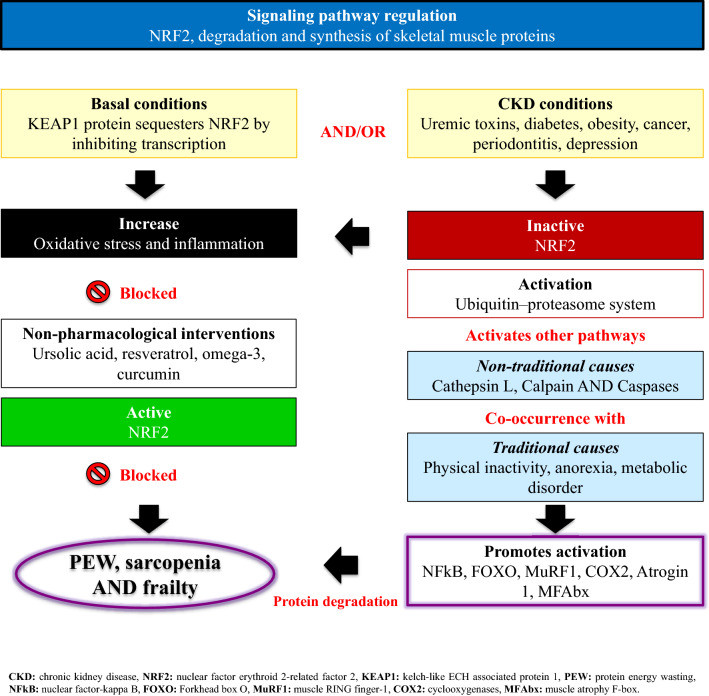

## Introduction

Muscle mass, which is the most abundant tissue in the human body, accounting for ~ 40% of body mass in young adults and 20–30% in older adults, plays, in addition to its role in body movement, including respiration, many other essential roles, e.g. in glucose homeostasis and as an endocrine organ that influences the health of the whole body [1]. Loss of muscle protein, leading to deterioration of muscle mass and muscle function, is the typical feature of sarcopenia linked to aging and physical inactivity, and of protein–energy wasting linked to chronic diseases such as cancer, diabetes and chronic kidney disease (CKD). These conditions, which are accompanied by increased muscle protein catabolism leading to muscle wasting, contribute to increased risk of hospitalization, morbidity and mortality in patients with CKD [2, 3].

Protein-energy wasting (PEW), usually defined as a pathological state with accelerated depletion of protein and fat deposits resulting in involuntary loss of > 10% of body weight in the absence of concomitant infections, tumor diseases or chronic diarrhea, is induced by factors such as inflammation and oxidative stress [2]. PEW is common in CKD, with a prevalence between 11% and 54% in CKD stages 3–5 and 28–54% in CKD stage 5 on dialysis [4], and is associated with complications that negatively impact on quality of life [5, 6] and survival [2]. Protein depletion, especially in skeletal muscle, in patients with PEW can not be prevented or reversed solely by increasing intake of protein and other nutrients, since the wasting process is also due to underlying metabolic changes rather than to insufficient nutrient intake alone [7, 8].

Sarcopenia is characterized by the concomitant conditions of both low muscle strength and low muscle mass, and  is a consequence of aging that predisposes to frailty; sarcopenia is common in CKD (and many other chronic diseases) especially in aging individuals, tobacco smokers and those with insufficient  food intake, neuronal damage or metabolic disorders [3, 9, 10].

In patients with CKD exhibiting PEW and/or sarcopenia, the development of muscle alterations, including low muscle mass and low muscle strength, depends on many underlying factors and pathophysiological mechanisms. Some of the most important ones are anorexia/reduced protein intake, physical inactivity, metabolic disorders (e.g. metabolic acidosis, electrolyte disorders, uremic toxin accumulation), hormonal disorders (e.g. insulin resistance, vitamin D deficiency, low testosterone), catabolic effects of dialysis*,* and persistent oxidative stress and inflammation, typically accompanied by increased circulating concentrations of tumor necrosis factor [TNFα], interleukin [IL] IL-6, IL-1, transforming growth factor-beta [TGF-β] and interferon-gamma [IFN-γ]) [2, 3], that, together with microRNA responses and dysregulation of angiotensin II, and cellular processes blocking myogenesis, further aggravate the catabolic state [11–15].

While to date no reliable therapeutic options are available in routine clinical care to prevent muscle wasting due to PEW in patients with CKD, the unraveling of catabolic pathways that govern protein turnover and degradation such as the ubiquitin–proteasome system (UPS) [11] and its links with potentially modifiable factors, such as the nuclear factor erythroid 2-related factor 2 (NRF2)-mediated antioxidant response signaling pathway, gives hope that strategies to suppress or block protein degradation could soon be within reach. Pharmacological treatment based on inhibition of the NRF2– kelch-like ECH associated protein 1 (KEAP1) signaling pathway has successfully been applied in treatment of diabetic kidney disease [16]. Non-pharmacological treatment strategies, including NRF2-induction by food components and dietary supplements, are gaining increased attention as part of the “food as medicine” concept, targeting the foodome (including > 25,000 substances that make up the human diet) [17].

In this brief review, we present recent developments in the research on the role of the NRF2-mediated antioxidant response signaling pathway for the maintenance of muscle mass in patients with CKD, through its impact on protein degradation by UPS and other mechanisms, and present examples of potential non-pharmacological interventions using nutraceutical activators to improve redox homeostasis, aiming at preventing skeletal muscle wasting associated with PEW in patients with CKD.

## Ubiquitin–proteasome system: NRF2 signaling pathway and its effect on muscle

**Fig. 1:**
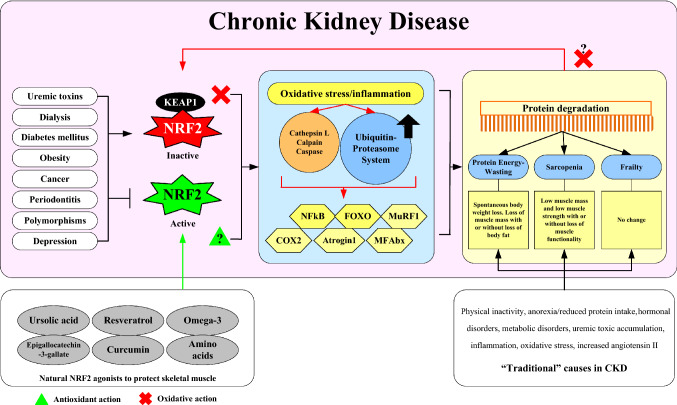
Schematic representation of factors promoting NRF2-deficiency in patients with chronic kidney disease and mechanisms by which this may result in protein-energy wasting (PEW) and muscle wasting, by promoting oxidative stress/inflammation and activating the ubiquitin–proteasome system (UPS) pathway

The etiology of pathophysiological mechanisms leading to muscle wasting is complex and includes mechanisms that increase muscular proteolysis through increased catabolism and apoptosis activation, and decrease the synthesis of muscle cells [11, 18]. Specific mechanisms, triggered by factors such as oxidative stress and inflammation [1, 11], include the caspase pathway, the lysosomal proteolytic system (cathepsin L), the calcium-dependent proteolytic calpain system [19], and not least the UPS, which are all strongly associated with PEW in CKD [2, 11] (Fig. 1). Through the insulin-like growth factor-1 (IGF-1)/phosphatidyl inositol-3 kinase (PI3K)/protein kinase B (Akt) pathway [20], one of the most explored anabolic signaling pathways affecting muscle in CKD, proteolysis in muscle cells occurs when there is a suppression of PI3-K activity in muscle that is induced, for example, by acidosis, leading to accelerated muscle proteolysis [20].

Depletion of antioxidant enzymes (among others, superoxide dismutase [SOD1, SOD2, SOD3], hemoxygenase [HO1, HO2], glutathione peroxidase [GPx], catalase [CAT], and glutathione [GSH]) [21] promotes mitochondrial dysfunction and increases oxidative stress, causing damage to multiple cellular components such as DNA, proteins, and lipids, in metabolic and chronic diseases [15, 22]. In general, oxidative stress with increased levels of reactive oxygen species (ROS, superoxide [O2-], hydrogen peroxide [H2O2], hydroxyl radical [-OH] and peroxynitrite [ONOO-]) is the result of enzymatic activity of the mitochondrial respiratory system, such as cyclooxygenases (COXs), cytochrome P450, and myeloperoxidases [23]. In addition, alterations in the activity of various transcription factors regulating oxidant and antioxidant genes, such as Forkhead box O (FOXO), nuclear factor-kappa B (NFκB) and NRF2 have been studied in murine models but less so in humans [15, 24]. A systematic review of the relations between NRF2 and morbidity in CKD was recently published [16].

In response to oxidative attacks, cells turn on antioxidant defense systems, such as the NRF2 system, to maintain cell redox homeostasis and protect cells [22], while UPS, the main regulatory mechanism of skeletal muscle degradation, is activated. UPS involves three enzymes: ubiquitin-activating enzyme E1; ubiquitin-conjugating enzyme E2; and ubiquitin protein ligase E3 that regulates selectivity and specificity of protein degradation mechanisms [25]. Two muscle-specific E3 ubiquitin ligases, muscle RING finger-1 (MuRF1) and muscle atrophy F-box (MAFbx; atrogin-1) increase transcriptionally in skeletal muscle under atrophy-inducing conditions. MuRF1 participates in the contraction and structure of muscle proteins, while MAFbx participates in protein synthesis and muscle regeneration, but by acting together with myostatin it may also have a role in protein degradation leading to atrophy of skeletal muscle [25]. UPS could be considered responsible for the control and balance of both anabolism and catabolism of skeletal muscle proteins in conditions, such as prolonged fasting, diabetes and cancer, which are accompanied by high mRNA levels of MuRF1 and MAFbx, by activation of the NFkB system and repression of NRF2 [25]. However, the situation in CKD is unclear as information is rather scarce and mainly based on animal models.

Data from experimental studies suggest that intracellular activation of the caspase-12, 9 and 3 pathways stimulate the production of ROS and activate NFκB and the nuclear factor of kappa light polypeptide gene enhancer in B-cell inhibitor, alpha [IkBα] [1, 26] to promote "ubiquitination" apoptosis and cellular autophagy; the latter prevents mitochondrial biogenesis at the muscular level by stimulating the degradation of cytosolic proteins (actomyosin) and organelles. These events are also highly regulated by the lysosomal pathway, in which the role of cathepsin L seems to be pivotal [2, 26]. Cathepsin L may also be present extracellularly, independent of the lysosomal fraction, playing a special role during atrophy [6, 27]. Also, calcium-dependent calpains (calpain 1, μ-calpain; calpain 2, m-calpain) are activated in hypoxic conditions [28], as well as in CKD associated with the induction of hypoxia factors 1-α, such as HIF1α, HIF2α, HIF3α which may contribute to muscle atrophy, muscle wasting and frailty [19] involving oxidation of contractile proteins, actin and myosin [26].

Furthermore, the COX pathway (especially COX-1 and COX-2) with synthesis of prostaglandins (PG, PGE_2_, PGF_2α_, PGI_2_ and PGD_2_) from arachidonic acid also regulates muscle regeneration and affects muscle degradation by modulating inflammation and myogenesis [29, 30]. COX-2/PGE2 responses induced by altered renal blood flow and pro-inflammatory cytokine activity contribute to the development of CKD, which associates with up to four-fold higher COX-2 than in absence of this disease, and to appetite loss and altered energy metabolism by blocking the central nervous system, thus promoting several pathways leading to PEW [31, 32]. Moreover, PGs derived from COX-2, but not from COX-1, are critical for muscle regeneration, which is consistent with their role in blocking repair of various systems and organs, including the kidneys [29, 30]. Notwithstanding, there are a limited number of studies exploring the role of NRF2 and UPS and the above mentioned factors for the associations of oxidative stress and inflammation with skeletal muscle alterations in CKD.

Similarly, the FOXO transcription factor is expressed in skeletal muscle in three main isoforms: FOXO1, FOXO3 and FOXO4. Evidence suggests that translocation of FOXO (especially FOXO1 and FOXO3) to the nucleus promotes increased expression of atrogin-1 (MAFbx) and MuRF1 (type E3 ligases) thus promoting muscle atrophy [1, 33]. Oxidative stress and inflammation [26] are regulators of the expression of MuRF1 and MAFbx via p38 mitogen activated protein kinase (MAPK), FOXO and NFkB [25].

The central importance of NFkB is highlighted by the fact that it regulates the expression of many genes including those responsible for muscle proteolysis. Activation of NFkB via the UPS and the MuRF1 pathways induces muscle degradation and muscle wasting, which in turn induces cellular apoptosis [19]. Higher levels of NFkB have been observed in hemodialysis patients with poor nutritional status, reinforcing the hypothesis that inflammation is a key driver of PEW [34]. Furthermore, blocking the translocation to the nucleus by pharmacological or genetic inhibition of NFkB prevents the expression of several components of the proteolytic machinery including MuRF1, muscle synthesis and preservation [6]. NRF2 is also influenced by the inflammatory cytokine tumor necrosis factor (TNF)-like weak inducer of apoptosis (TWEAK), and as NRF2 activation inhibits TWEAK-induced atrophy in myotubes, NRF2 may protect skeletal muscle from TWEAK-induced cell death [35].

NRF2 protein (605 amino acids, belongs to a subset of the basic leucine zipper family proteins) is responsible for antioxidant transcription (as a master regulator) and, under normal conditions, it is bound to KEAP1 [36, 37]. This ubiquitin conjugation, NRF2-KEAP1, favors rapid proteasome degradation in the cytoplasm by activating the ubiquitin ligase complex Cul3-E3; however, in a state of stress, NRF2 is released from KEAP1 and rapidly accumulates in the nucleus, activating the antioxidant response element (ARE) in the promoter region of many antioxidant genes [22], which in turn leads to increased regulation of antioxidants and phase II detoxifying enzymes [36]. Nuclear respiratory factor 1 (NRF1) contains multiple RNAs in the promoter part of its gene, which are necessary to promote NRF2 activity from the induction of ROS [38]. Thus, NRF2 is sensitive to redox state and plays a role in the regulation of UPS components [39]; therefore, it modulates pro-apoptotic signals, such as NFkB, ASK1, BAD, BAX, AIF, AP1, peroxisome proliferator-activated coactivator gamma 1-alpha (PGC1α) and caspases 9 and 3, but the response depends on the accumulation of ROS and depletion of GSH [18].

The effect on skeletal muscle remains poorly understood; whereas higher NRF2 expression has been observed in non-dialysis CKD patients, hemodialysis patients exhibited reduced NRF2 gene expression, which was associated with increased NFkB gene expression possibly related to systemic inflammation. Considering that the uremic milieu in CKD patients (CKD stage 4) has been associated with up-regulation of NRF2 [40], it is of interest that a recent investigation evaluating the humanin peptide and the mitochondrial open reading frame of 12S rRNA-c (MOTS-c) related to cell survival, suppression of apoptosis in oxidative stress or starvation, as well as enhanced insulin secretion and action found that patients with stage 5 CKD had increased circulating levels but reduced local muscle expression of humanin [41]. On the other hand, in CKD stage 5 patients, MOTS-c levels were observed to be reduced in both serum and muscle, together with a reduction of NRF2 expression in muscle [41]. Protein degradation in skeletal muscle has been scarcely described in relation to this pathology [42]. However, a study in older adults—presumably with some degree of aging-related sarcopenia—reported lower expression of NRF2 [43]. This might affect redox homeostasis and alter skeletal muscle structure and function through altering the balance between oxidizing and antioxidant agents [18, 44].

While clinical and experimental data suggest that hyperphosphatemia, a prominent alteration in CKD, accelerates muscle wasting, the underlying mechanism remains unclear. However, data in mice suggest that hyperphosphatemia suppresses myogenic differentiation in vitro and promotes muscle atrophy in vivo through oxidative stress-mediated protein degradation and both canonical (ROS-mediated) and non-canonical (p62-mediated) activation of NRF2 signaling [45].

In general, myocytes in skeletal muscle are enriched with mitochondria, that could account for as much as 1/3 of the total weight of the cell, facilitating the excessive production and accumulation of ROS, dependent on CKD stage and age [15, 18, 44]. NRF2 deficiency may exacerbate age-related mitochondrial oxidative stress in aged skeletal muscle [46] and be part of an intermediate inflammatory phenotype that promotes burden of lifestyle diseases that accumulate with age [47]. The decrease in the number of fat-free myocytes and the low transcription of NRF2 [44] may be related to several factors, such as inadequate diet, hyperparathyroidism, depression, dementia, osteoporosis, periodontitis [43], dialysis, uremic toxins [48], obesity, hyperglycemia, variants of *NFE2L2* gene (encoding NRF2 protein) [22, 49], metabolic acidosis and endothelial dysfunction [23, 34]. All these factors promote muscle degradation and may contribute to skeletal muscle dysfunction by inducing ubiquitination, lipid peroxidation and activation of apoptotic processes and autophagy with mediators such as calpain and caspases [18]. Moreover, although little is described in humans, the catalyzed reaction of COX appears to contribute to PEW through the NRF2 signaling pathway [14, 18]. The NRF2-related molecular mechanisms leading to muscle dysfunction have not been fully described; new lines of study are open on how NRF2 dysregulation affects muscle mass, quality and function, and leads to PEW in the context of CKD [50].

A better understanding of these processes is of importance to design therapeutic strategies to reverse these complications. To date, there is, with few exceptions, a lack of studies clinically demonstrating the safety and effectiveness of drugs targeting NRF2 to address these complications in humans [51]. A clinical trial in patients with obesity, diabetes, and stage 4 CKD who received bardoxolone methyl demonstrated improved glycemic control, weight loss, decreased lipid accumulation, and reduced inflammation through activation of NRF2 and inhibition of NFkB [40]. On the other hand, a growing number of studies have explored non-pharmacological approaches to enhance NRF2-related mechanisms affecting protein synthesis and degradation to prevent muscle proteolysis or promote muscle synthesis, including resistance training [38]; however, in a study conducted in NRF2-deficient mice during endurance exercise stress, it was proposed that other NRF2-independent mechanisms such as PGC1α are activated by inducing mitochondrial biogenesis in aging skeletal muscle cells [18], which may also be activated by derivatives or food supplements known as nutraceuticals [14, 52].

## Nutraceuticals: activation of NRF2 to protect skeletal muscle against PEW

In recent years, many dietary components, plants and extracts have been found to promote NRF2 activation, such as sulforaphane, curcumin, resveratrol, lycopene, capsaicin, chlorogenic acid, fish oil, bilberry pomace, coenzyme Q10, broccoli sprouts, cherry juice, and grape polyphenols [53, 54] showing different structure and action mechanisms in human intervention trials [54]. To date, there is no study in CKD describing the signaling pathways blocking NFkB-activation of NRF2, but it is believed that the effect is mediated by regulation of the enzymes MuRF1 and MAFbx (atrogin-1) in the UPS; these pathways are induced in response to oxidative stress and inflammation, and improve mitochondrial function [37, 50, 51]. Therefore, targeting the NRF2 pathway with rationally designed nutraceuticals could be a promising therapeutic approach, and some studies have attempted to identify the mechanisms underlying NRF2 activation by dietary compounds, and to establish their protective effects in skeletal muscle [55]. The following is a description of some examples of nutraceuticals.

### Epigallocatechin-3-gallate (*Camellia sinensis*)

Epigallocatechin-3-gallate (EGCG), the main phenolic component of green tea (containing ∼77.8 mg EGCG per gram of dried leaves) [56], is considered a potent agent to counteract cachexia in cancer patients [57]. This polyphenol has also been suggested to be useful in the prevention and treatment of CKD [56]; however, the mechanism by which EGCG improves kidney function has not been clarified. EGCG is known to be a potent NRF2 activator and its possible action is centered on the modification of critical cysteine residues contained in KEAP1, which favors dissociation of the KEAP1-NRF2 complex or increases the stability of NRF2 [58]. This effect of EGCG could play a role as a treatment of PEW in patients with CKD, since its antioxidant action has been associated with prevention of unintentional weight loss; EGCG attenuates leukocyte infiltration into the skeletal muscle by decreasing NFkB, MuRF1 and MAFbx, thereby reducing the inflammatory activity and favoring regeneration of muscle fibers [50]. Furthermore, EGCG had positive effects on decreasing apoptosis of skeletal muscle tissues, relieving oxidative stress damage and suppressing inflammatory cytokine production; EGCG also had positive effects on the activation of the NRF2/HO-1 signaling pathway [55].

### Resveratrol

Resveratrol, a phenolic compound, has anti-inflammatory and antioxidant effects favoring the secretion of a greater amount of antioxidant enzymes, and promotes the activation of transcription factors such as NRF2 [59]; therefore, it can prevent protein degradation (including cachexia and muscle atrophy) mainly induced by angiotensin I and II pathways, phorbol ester, 12-O-tetradecanoylphorbol-13-acetate (TPA), by downregulating the expression of NFκB, MuRF1 and MAFbx [6]. In addition, increased NRF2 transcription has been associated with modulation of proinflammatory cytokines (mainly TNF and IL-6) [60], and furthermore, activation of NRF2 and Sirtuin 1 (SIRT1) signaling pathways were reported to ameliorate mitochondrial dysfunction [61], and to improve kidney function, proteinuria, histological changes, and inflammation in aged mice [61]. However, there is controversy about the final effect since some studies reported that 100 mg/day did not modify muscle mass [60] and 500 mg over 4 weeks did not have an antioxidant or anti-inflammatory effect in patients with predialysis CKD [59]. More studies are needed on the potential effect of resveratrol on PEW in patients with CKD.

### Amino acids and carbohydrates

Increased intake of proteins, amino acids and carbohydrates improves nitrogen balance and overall has a positive effect on muscle mass, but due to a possible influence on the expression of E3 enzymes of the UPS this relationship is complex. An experimental study showed that a diet low in protein and high in carbohydrates decreased muscle protein reserves and availability of amino acids while muscle proteolysis decreased linked to reductions in the mRNA levels of atrogin-1 and MuRF1, ubiquitin conjugates, proteasome activity, and activity of caspase-3 [62]. The decrease in muscle proteolysis was thought to represent an adaptive response to spare proteins in a condition of diminished availability of dietary amino acids. Contrary to expectations, dietary protein supplementation (∼20 g twice daily) did not attenuate muscle loss during short-term muscle disuse in healthy older men possibly because there was an increase in mRNA expression of MurF1 and MAFbx [63]. Nevertheless, supplementation of specific amino acids may have a beneficial effect on muscle mass; for example, leucine supplementation attenuates muscle wasting induced by immobilization by minimizing gene expression of E3 ligases, which consequently could downregulate UPS-driven protein degradation and may be related to NRF2 activation [64, 65]. In addition, a combination of leucine, valine, and isoleucine reduced the expression of MAFbx mRNA and prevented the increase in MuRF1 total protein in both resting and exercising muscle in humans [66]. Other studies suggest that glutamine supplementation in diabetic rats is potentially useful for slowing the progression of muscle atrophy and muscle wasting [67]. While increased intake of proteins, amino acids and carbohydrates in general can be expected to have a positive impact on muscle mass in patients with CKD, clarification of the effects related to the interplay between NRF2 and UPS are warranted.

### Omega-3

The role of different dietary fats on muscle metabolism and regulation, specifically of E3 enzymes, has been evaluated in several studies, but none in CKD. In vivo studies have described the upregulation of *NRF2* gene after supplementation with 2700 mg/day of omega-3 in patients with diabetes due to the antioxidant effect of NRF2 [68]. It has also been described that omega-3 is related to mitochondrial biogenesis, which favors NRF1 and NRF2 [69], an effect attributed to greater expression and deacetylation of PGC-1, which is triggered by SIRT1 [69]. In this sense, rats with cancer cachexia receiving omega-3 showed an increase in MuRF1 and MAFbx and a decrease in the proinflammatory cytokine TNF-α [8]. However, studies on the impact of various nutritional factors on muscle metabolism and regulation of MuRF1 and MAFbx by blocking NRF2 activation by NFkB (mainly investigated in animal models and in vitro studies) have shown inconsistent results.

### Ursolic acid

Ursolic acid is a lipophilic pentacyclic triterpenoid derived from apple peels, basil (*Ocimum basilicum*), blueberry (*Vaccinium* spp), cranberry (*Vaccinium macrocarpon*), heather flower (*Calluna vulgaris*), Labrador tea (*Ledum groenlandicum Retzius*), olive (*Olea europaea*), pear (*Pyrus pyrifolia*), and rosemary (*Rosmarinus officinalis*) [70]. Benefits attributed to ursolic acid include limiting ROS production, lipid peroxidation and DNA damage in human keratinocyte HaCaT cells [71]. In addition, ursolic acid was recently reported to protect the brain against ischemia and to protect the liver against CCl4-induced damage in mice via the NRF2 pathway [72]. Furthermore, ursolic acid may block CKD-induced loss of muscle mass by reducing the expression of myostatin and pro-inflammatory cytokines, stimulating IGF-1 signalling, and reversing insulin resistance [70]. Consumption of ursolic acid was reported to reduce muscle atrophy in mice by decreasing the expression of MuRF1 and MAFbx [71]; in CKD patients, further studies are needed to assess its efficacy for preventing muscle wasting.

### Curcumin

Curcumin, which is the active ingredient of turmeric (*Longa Turmeric*) that is widely used as a food ingredient in several Asian countries, has been reported to have positive effects in conditions such as cancer, Alzheimer’s disease, and ulcerative colitis, as well as in athletes. Curcumin has been shown to modulate expression of various genes with anti-inflammatory activity thereby reducing TNF, IL-8, IL-1β, COX-2 [73], kaveolin-1 [Cav-1] and NFkB [74]. Curcumin has anti-proliferative effects as it decreases over-expression of monocyte chemotactic protein-1 [MCP-1], reducing the risk of renal fibrosis, and antioxidant effects because it induces expression of HO and NRF2 [31]. Interestingly, curcumin prevents muscle proteolysis by decreasing the expression of E3 ligases, MuRF1 and MAFbx (atrogin-1) [75] through mechanisms dependent on NFkB, thereby inhibiting UPS activity [75]. Thus, curcumin’s ability to enhance the redox state in cells may produce a beneficial effect on muscle function [24, 74]. In addition, some studies suggest that skeletal muscle changes are possibly due to low NRF2 activity, and this in turn favors and potentiates oxidative stress and compromises antioxidant capacity [24]. Curcumin modulates the antioxidant response through NRF2, increasing HO1, SOD, GPx and GSH, and decreasing the expression of oxidative stress mediators by acting on NFkB. Consequently, curcumin has the ability to accelerate muscle biogenesis and regulate the NFkB and NRF2 pathways [76].

Experimental studies have described the effect of curcumin on inhibition of COX-1 and COX-2 expressed in mesangial cells and macrophages [31, 32]. COX activity blocks muscle regeneration, but this is easily inhibited by the administration of drugs and/or nutraceuticals with antioxidant properties, such as curcumin [29, 73]. In this sense, by mediating the COX-2/PGE2 pathway, administration of curcumin could be a potential therapeutic intervention in patients with CKD, not only in the treatment of muscle wasting, but also in the prevention and progression of renal damage [5, 32]. Therefore, further research in this area is of importance to identify the specific dose and type of curcuminoid to be used in order to achieve benefits while avoiding high doses that could have pro-apoptotic effects [76, 77].

Curcumin significantly inhibits proteolytic pathways such as cathepsin L and calcium-dependent calpain pathway, implying that curcumin could inhibit multiple proteolytic pathways through a direct effect on muscle catabolism [75]. Experimental studies in rodents, as well as in the general population, suggest possible action of curcumin against muscle wasting, sarcopenia and frailty [75, 76]; however, a controversial study in advanced pancreatic cancer patients showed that 8 g/day of curcumin orally administered for 2 months, decreased fat and muscle mass [77]. The complexity of the patients evaluated in the latter study appeared to be a confusing factor, due to poor bioavailability and rapid metabolism [32]. Finally, it should be mentioned that it has been difficult to extrapolate the appropriate curcumin dose in humans from studies in murine models.

Finally, it is important to consider that other pathways have been described that may likely be involved, for example, recently a study conducted in rats induced with CKD found that muscle protein synthesis can be increased without depending on a higher intake of nutrients or with an increase in exercise but associated with the elimination of nucleolar protein 66 from the body [78]. However, it is important to highlight that in CKD, strong activation (early nephropathy) or repression (advanced nephropathy) of endogenous NRF2 can be generated and depends on the cause, comorbidities, stage and duration of CKD, as well as on the accumulation of uremic toxins. Therefore, therapies targeting the NRF2 system will need to focus on a diversified approach that allows NRF2 to be increased or decreased according to homeostatic requirements [79].

## Conclusions and perspectives

Loss of muscle proteins leading to deterioration of muscle mass and muscle function are typical features of protein–energy wasting and sarcopenia in patients with CKD. Inflammation and oxidative stress are key drivers of processes leading to muscle wasting, by activating catabolic pathways governing protein turnover and degradation such as the UPS, a main regulatory mechanism of increased muscle protein catabolism. Based on available data, we hypothesize that muscle wasting in patients with CKD could be mainly due to deficiency in NRF2 transcription, in combination with inflammation and increased oxidative stress, and that transcription factor NRF2 may represent a potential therapeutic target for preventing muscle wasting. This hypothesis is supported by experimental studies in animal models showing that the decrease of NRF2 alters several signaling pathways associated with regulation of protein turnover in skeletal muscle. We explored whether non-pharmacological interventions with nutraceuticals containing NRF2 inducers to improve redox homeostasis could be an option to prevent muscle wasting in CKD. The analysis of the different pathways that participate in protein synthesis is a new line of research, to be further explored in patients with CKD.
